# With a little help from my ‘ordinary friends’: relationships, networks, and resilience in Masisi, North Kivu, eastern Democratic Republic of the Congo

**DOI:** 10.1111/disa.70055

**Published:** 2026-05-10

**Authors:** Solange G. Fontana

**Affiliations:** ^1^ NIOD Institute for War Holocaust and Genocide Studies the Netherlands

**Keywords:** civilian protection, civilian protective agency, conflict‐affected area(s), coping strategies, grassroots associations, informal social protection, living with war, resilience in/to conflict, resilient communities, resisting war, social connections/networks/relations, zones of peace

## Abstract

Are social networks the key to understanding resilience in conflict? Recent studies suggest so, but relational research in conflict‐affected areas is rare. What exists stresses the importance of small circles of close family members, trusted friends, and co‐ethnic persons/groups, but tends to overlook their aggregate effect. Drawing on data from a relational study of personal support networks in Masisi, a rural area of North Kivu province, eastern Democratic Republic of the Congo, I show how people rely on large, widespread networks of ethnically diverse recent strangers. I highlight the types of relationships people value most, assess how they make and maintain them, and begin to explore some of their cumulative socio‐political effects. I posit that reputation and improved local security are support networks' most important outcomes, although the latter comes at a price. The findings reveal a fundamental paradox: while resilience in conflict is primarily relational, its dependence on connections limits its scope and makes it inherently unstable.

## 1 INTRODUCTION

Sifting through a friend's wedding photographs, I commented on the number of elegantly dressed chefs. He laughed and told me: ‘indeed! The whole neighbourhood turned up to cook!’. In Masisi, a territory of North Kivu province in the beleaguered east of the Democratic Republic of the Congo (DRC), people regularly ‘turn up’, and not just to cook for weddings. People in Masisi (or Masisiens) are supported by large, widespread networks of ethnically diverse friends, neighbours, acquaintances, and recent strangers, who they call ‘ordinary friends’. These circles of support do not represent their *full* social networks; rather, they are a subset, composed of the everyday relationships that people rely on most to negotiate the vagaries of living with poverty and recurrent conflict.

Masisi is a difficult place. The territory has experienced outbreaks of violence since at least the nineteenth century (Stearns, 2012; Mathys, 2021). Today, its people juggle the difficulties of living with endemic poverty and the exactions of an array of ever‐shifting armed groups, including those of the Rwanda‐supported March 23 Movement (M23). The violence is the product of intersecting colonial histories and post‐colonial inequities, overlaid with regional and international dynamics (Stearns, 2021; Mathys, 2025). The aftermath of the 1994 genocide of the Tutsis in neighbouring Rwanda overwhelmed the fragile Zairean state,^1^ culminating in the First and Second Congo Wars in 1996–97 and 1998–2003, respectively (Prunier, 2008; Lemarchand, 2009). This was followed by almost two decades of violent peace, marked by everyday exactions, small‐scale armed group activity, and two Rwandan‐backed insurgencies—by the CNDP (National Congress for the Defence of the People) in 2006–09 and by M23 in 2012–13 (Berwouts, 2017). In 2022, M23 renewed its offensive. By 2025, conflict had displaced an estimated 2.3 million people across North Kivu province (UNHCR, 2025). These cycles of violence have taken a heavy toll on Masisi's people. Deaths of family members and repeated displacement are depressingly common (Vink and Pham, 2013–20). At the same time, Masisiens contend with widespread poverty, extensive corruption, weak rule of law, poor infrastructure, a lack of services, and land expropriation by urban elites (Mararo, 1997; Stearns, 2012; Vink and Pham, 2013–20). When describing how they negotiate the difficulties of such an environment, Masisiens highlight the importance of certain types of relationships and networks, and it is these, along with their consequences and cumulative effects, that I explore in this article.

Although the literature on support networks in conflict suggests that people rely most on family members and co‐ethnic persons/groups, I argue that for everyday assistance and protection, Masisiens depend on large, geographically widespread, ethnically diverse support networks composed primarily of friends, neighbours, and acquaintances, many of them recent strangers. As noted, Masisiens call these people ‘ordinary friends’, differentiating them from their one or two ‘intimate friends’. This does not mean that they ignore family and co‐ethnics—they may turn to them for support—but they are not necessarily their first recourse. War and poverty have taken their toll on such ties, while weakened family authority combines with polygamy and inheritance disputes to strain kin relations.

Masisiens believe that certain types of relationships make receiving assistance/protection more likely. Hence, they deliberately seek them out, developing key network traits in the process—that is, many members, a wide geographical spread, and an ethnically diverse composition. These traits reflect Masisiens' perceptions of their principal risks: poverty, mobility,^2^ and ethnically‐targeted exclusions and violence. Importantly, though, Masisiens not only depend on their ordinary friends for social and physical protection, but they also use these relationships to make, maintain, and broadcast reputation. In Masisi, reputation has become a critical, de facto currency, ‘spendable’ on a wide range of goods and services.

Yet, Masisiens' support networks mainly include their socio‐economic peers, and therein lies the catch. While these networks just about create a buffer, their members usually lack the social and/or economic clout needed to make them reliably effective or sustainable. Resilience in Masisi may depend on people's connections, but it is the almost sole reliance on socio‐economic peers that limits scope and makes resilience inherently unstable. For many Masisiens, the most they can hope for are ephemeral moments of resilience. Sustainable resilience remains a chimera.

This article also begins to analyse some of the cumulative socio‐political effects that support networks produce as they intersect and criss‐cross communities and geographical space. In Masisi, support networks allow for validated information exchange. This combines with Masisiens' dependence on reputation to turn support networks—and their leaders—into important sources of influence and alternative governance. Community leaders leverage this aspect to govern the community and improve internal security. They also use community members' support networks, along with their own connections, to help prevent/limit the encroachment of violence from outside. Data suggest that while community leaders are relatively successful in governing internal security, staving off the encroachment of external violence should not be taken for granted. It is important to underscore that the modicum of governance and localised security that support networks allow comes at a price. Success depends on high levels of coercive social control. This, in turn, cements local hierarchies in ways that reinforce the very status quo that makes these types of support networks necessary in the first place.

Such findings add to literature that situates resilience in and to conflict as primarily relational, but this article goes further by seeking to explain why this is the case. In doing so, it extends current understandings of networks and social relations' role in informal social protection, as well as individual and community coping in situations of conflict. It nuances literature on resilience in conflict by identifying the types of relationships people perceive as assistive and/or protective, highlighting the role of reputation, while explaining support networks' limitations, including that stemming from its relational character. Moreover, this article furthers relational research into living with violence by beginning to probe some of support networks' cumulative socio‐political effects. By so doing, it illustrates how the international development/humanitarian literature on coping and social protection in conflict intersects with recent studies on civilian self‐protection, protective agency, and zones of peace, in ways that reveal enticing avenues for additional research.

### 1.1 Background: living with war? Resilience, social networks, and conflict

Resilience has become both a central humanitarian concept and elusive policy objective (Brassett, Croft, and Vaughan‐Williams, 2013, p. 221; Scott‐Smith, 2018; European Civil Protection and Humanitarian Aid Operations, 2025). While practitioners consider resilience to be a positive policy outcome, scholars note its political pitfalls (Duffield, 2012; Evans and Reid, 2013; Chandler, 2014). By the late 2010s, some scholars suggested that resilience was no longer a relevant policy framework (see, for example, Chandler, 2019). Nonetheless, it continues to be conceptually relevant in the DRC, not least as a descriptor of what people themselves say they strive for.

In this article I understand resilience in two ways and propose an addition, drawn from interviewees' own interpretation of the concept. The first is most familiar to emergency livelihood practitioners: ‘the ability of an individual, a community or a country to cope with, adapt and recover … from the impact of a disaster, violence or conflict’ (European Civil Protection and Humanitarian Aid Operations, 2025). This is resilience *in* conflict. Resilience *to* conflict, meanwhile, is the ability of an individual, household, or community to reduce the impact of violence and/or prevent it encroaching from outside, even temporarily (Krause et al., 2023). Such definitions explain resilience as the ability to adapt, but what allows adaptation remains undefined. Building on my own research and studies of grassroots understandings of resilience in Kenya's conflict‐prone northern highlands, I posit that resilience in conflict is synonymous with flexibility (Semplici, 2019; Fontana, 2020; Semplici and Campbell, 2023), and that people's support networks are their safest, most *flexible* asset. Thinking about resilience as flexibility reconciles the two different understandings of resilience, while emphasising its relational nature. But resilience's reliance on relationships is double‐edged. If on the one hand connections are people's safest, most flexible asset, they cannot always deliver the material and financial support people need, while changing circumstances mean that today's valued relationship can become tomorrow's liability. Furthermore, peoples' support networks are shaped by the socio‐political environment in which they are embedded. Thus, even when short‐term outcomes are positive, longer‐term and cumulative outcomes may be less so.

Violent conflict is a depressing feature of the contemporary humanitarian landscape. Its obstinacy has heightened interest in how people live in such environments and the past 15 years have seen several large, multi‐year research projects designed to inform policy and practice. Take the Secure Livelihoods Research Consortium, an eight‐year, collaborative project that brought together data from eight African and Asian countries, including the (eastern) DRC. While not explicitly relational, its reports repeatedly note how people living ‘amid violence’ lean on their relationships and networks (Schomerus, 2023). Similarly, in 2017, the Feinstein International Center at Tufts University reviewed 15 years‐worth of livelihoods data from conflict‐affected areas. Its conclusion was that resilience in conflict is underpinned by *social connectedness*, a concept that encompasses individual social relations and the wider networks they compose (Maxwell et al., 2017). Building on this insight, the Feinstein International Center partnered with Mercy Corps to investigate the relationship between social connectedness, informal social protection, and resilience (Kim, 2020; Stites and Humphrey, 2020; Stites, Humphrey, and Krystalli, 2021; Kim et al., 2022). These endeavours underline how relationships provide informal social protection in ways that can bolster or undermine resilience. Importantly, they are among the few initiatives that begin to deconstruct peoples' support networks.

Although these are crucial facets of resilience, this livelihoods‐based understanding of resilience is not the full picture. Scholars interested in individual and community self‐protection and grassroots peace also highlight the importance of connections (Baines and Paddon, 2012; Kaplan, 2017; Krause, 2018; Mac Ginty, 2021; Howe, 2023; Krause et al., 2023). For them, resilience in conflict includes the ways in which people and communities seek to lessen the impacts of violence around them (Krause et al., 2023, p. 5). This is done individually and communally. At an individual level, it amounts to the ability of social networks to mitigate threats and harms, such as beatings by armed actors, forced enrolments, arbitrary arrests, and sexual violence. At a communal level, it describes the capacity to resist internal violence and stop its external encroachment. This too is heavily dependent on social relations, both directly—the relationships on which community leaders draw—and indirectly—via the peer pressure created and exerted by the criss‐crossing webs of people's relationships.

The importance of social relations is also noted by conflict researchers who find that in (ethnic) conflict, people tend to rely most on restricted circles of close family and co‐ethnics (Vermeersch, 2011; Maxwell et al., 2016, 2017; Tilly, 2017; Marks and Stys, 2019). To explain why this is the case, scholars often conceptualise these connections as *strong ties* or *bonding social capital*. Tendentially *strong ties* are between people who are socially similar and whose social circles overlap. These factors help to ‘bond’ people, reinforcing trust—a commodity that is in short supply in conflict‐affected areas. Interestingly, few studies document the importance of friends or acquaintances—a notable exception being Nguya (2019). Those that do, explain their finding by emphasising the ‘strength of weak ties’ (Granovetter, 1983). *Weak ties* are friends with whom we are less involved, and acquaintances. Such people have different social circles and are often socially different in some way. This allows such relationships to ‘bridge’ social worlds and connect people to goods otherwise beyond their reach. Researchers posit that such linkages provide the most effective support.

Although a wide variety of case studies across disciplines find that relationships and networks are crucial to those living with conflict, most of what we know is culled from ‘post hoc network constructions of data collected for other purposes’ (Stys et al., 2022, p. 241). Explicitly relational research in conflict remains rare.

## 2 METHODOLOGY

This article is based on data from a relational, mixed‐methods study of people's everyday support networks carried out in Masisi's villages, internal displaced persons (IDP) camps, and principal towns between 2014 and 2020. The study is based mainly on semi‐structured interviews (key informant interviews with individuals, interviews with association members, household interviews, and individual life histories), some focus group discussions (FGDs), and archival work at the Institut Supérieur Pédagogique (ISP) de Bukavu.

In total, 433 women and men aged between 16 and 92 years participated in 262 semi‐structured interviews. Most of the interviews and FGDs were held in Masisi, but some took place in Goma, North Kivu's provincial capital. While I conducted the majority of the interviews with the help of Congolese research assistants, 80 household studies and 35 life histories were collected by two teams of Congolese researchers. I collaborated with Congolese researchers for a number of reasons (see below), but chief among them was the desire to put participants at ease and allow them to express themselves in the language that came easiest.^3^

Key informant, household, and association interviews and FGDs took between one and four hours to complete. Most were one‐off interviews, but a minority took place over several meetings (counted here as one interview). By contrast, the 35 life histories were considerably longer, averaging 6–10 hours each. These spanned multiple days, with researchers accompanying participants as they went about their daily lives.

Participants were initially introduced to us formally by authorities or informally by their friends. Further participants were recruited through snowball sampling. Depending on the type of interview, participants were purposefully selected by sex, age, current residential location, access to land, self‐reported principal livelihood or ethnic affiliation, and willingness to take part in a study involving repeat visits. For the association‐focused interviews, membership of, although not necessarily participation in, an association was an important criterion, as was deliberately choosing not to join an association or to withdraw from one. Of the participants in the household and life history interviews, 46 per cent were women and 54 per cent were men; the ethnic balance closely reflects that of the population‐based panel surveys of the Harvard Humanitarian Initiative (HHI) (Table 1).^4^

**TABLE 1 disa70055-tbl-0001:** Percentage of interviewees by ethnic group in comparison with HHI data.

	HHI baseline survey, 2014 Masisi sample size: 2010	115 qualitative interviews Household studies and life histories, 2015–16	HHI poll survey, 2017 Masisi sample size: 2018
**Hunde**	55%	**50%**	47%
**Hutu**	36%	**43%**	44%
**Tutsi**	3%	**3%**	3%
**Nande**	1%	**1%**	0%
**Nyanga**	1%	**1%**	0%
**Other**	4%	**2%**	6%

**Source:** author, compiled using own data and HHI data on http://www.peacebuildingdata.org/studies (last accessed on 24 April 2026).

For interviews not in French or English (the majority), I drew on the support of three capable research assistants. Additionally, I worked with five staff members of a Masisi‐based non‐governmental organisation (NGO), Blessed Aid, to secure the initial household interviews. For the life histories, I collaborated with seven graduate students of Masisien descent studying at the Université Libre des Pays des Grands Lacs (ULPGL) in Goma.

To work closely with a number of Congolese colleagues was a deliberate methodological choice. Their different strengths and perspectives helped to mitigate researcher bias, including that arising from my position as a privileged white woman from the Global North, as well as that of a former humanitarian worker—I worked in the area for almost five years before returning as a researcher. My Congolese colleagues' ability to navigate the social complexities and languages put participants at ease. Proof of this lies in the richness of their data. Daily exchanges with the interview teams, joint analysis sessions, and close collaboration during transcription and translation nuanced our understandings of the emerging stories. Nevertheless, all may not agree with my final analysis, and any analytical flaws are my own.

I complemented the interviews with fieldnotes, oral village/camp histories, and data from three days of 12‐hour observations with a small number of IDP households in the study area. The latter was generously shared by International Alert and provided valuable insights into the daily rituals in which people engage to build trust and maintain/extend support networks. I also triangulated the findings with relevant grey literature and HHI's population‐based panel surveys (2014–19). These track Masisiens' changing perceptions of social relations, including association membership. Additionally, 81 unpublished MA (Master of Arts)‐ and BA (Bachelor of Arts)‐level theses on various aspects of social relations, written by Congolese students at the ISP between 1973 and 1993, allowed me to trace the evolution of different types of support over time and contextualise them historically. The study was an iterative one, with successive periods of fieldwork building on emerging findings. With the help of MAXQDA content analysis software, I analysed the data relationally, identifying themes as they materialised.

Lastly, I am conscious that my findings differ from studies that highlight how in (ethnic) conflict, social networks constrict, with people relying most on family members and small circles of trusted friends and co‐ethnic persons/groups. These differences are possibly due to a combination of methodology and context. Most studies of coping strategies, support networks, or civilian protection do not take the relationships themselves as the primary unit of analysis. Rather, their starting point is the outcome—that is, social or physical protection. Furthermore, such research tends to be informed by one‐off interviews and/or surveys. As sociologist Matthew Desmond (2012, p. 1302) found, these tend to reveal the networks on which people *think* they rely, instead of those they use in practice. When I compared my own one‐off interviews with the information from people's life histories collected over repeat visits, I noted a similar phenomenon. Regarding contextual specificities: Masisi, and the DRC more broadly, have a long history of ethnically‐targeted exclusions and violence. These carry‐over effects of colonial debris overlay longer more nuanced stories of migration, cohabitation, integration, and, sometimes, conflict (Mathys, 2025). Today, Masisiens continue to make friends with ethnic ‘others’, and have come to view such relationships as protective. This attitude seems to be reinforced by the oft‐indiscriminate violence unleashed by Masisi's armed groups and their constantly shifting ‘unnatural’ alliances. Such behaviour undermines their rhetoric of ethnic self‐defence. For many of my research participants, armed groups are nothing more than self‐seeking “bandits”.^5^

## 3 SUPPORT NETWORKS AND RESILIENCE *IN* AND *TO* CONFLICT

### 3.1 Personal support networks and informal social protection

‘No *normal* person can be without friends! They improve your life, put food on the table, provide “insurance”, and improve security’.^6^ Indeed, to be friendless in Masisi is a catastrophe. Being friendless is how people describe poverty, while internal displacement is understood as moving to ‘where no one knows you’.^7^ ‘Wherever I end up I try to make friends quickly, these are the people who will help me’, one man explained.^8^ Masisiens fear of ostracism and its consequences make it an effective tool of social control, whether through peer pressure or when wielded deliberately by community leaders.^9^

#### 3.1.1 From family to friends

It is no longer the case that Masisiens turn to family (immediate and extended) for assistance, moral authority, and internal governance (Mararo, 1990). Today, ‘family is no longer enough’, and Masisiens rely most on a mixture of friends (43 per cent), fellow church members (37 per cent), and neighbours (33 per cent), with immediate and extended family mentioned only 22 and 24 per cent of the time, respectively.^10^ Moreover, in an area awash with aid, few people rely on NGO support (three per cent).^11^ The roots of this shift lie in the colonial reconfiguration of Congolese society (Young, 1966, p. 37; Fairhead, 1990, pp. 155, 326–331). This impoverished families, encouraged migration (for reasons of work, health, education, and/or conflict avoidance), and strengthened state‐sanctioned customary authority. The violence that began in the late 1980s only accelerated the change. Extended families (lineages) in Masisi now no longer have the land or wealth to assist their members or provide young men with land and a dowry. This deprives the youth of important rites of passage, which in turn has reduced the authority and social control of family heads. Furthermore, poverty and violence mean that people in Masisi move frequently, scattering families. Lastly, polygamy aggravates the situation. Indeed, in land‐poor Masisi, land inheritance is a major source of conflict. One man stated:

*Family members can be jealous and wish you ill… My brothers sold my eucalyptus trees without permission… Now they refuse to share the profits!* ^12^

Most of my interviewees reported that when it comes to assistance and moral support, friends, not family, are the safer, more reliable bet.

Masisiens divide friendships into the categories of ‘intimate’ and ‘ordinary’. Most have only one or two ‘intimate’ friends, in whom they confide unreservedly and share without expecting anything in return. Network literature describes such relationships, as alluded to earlier, as *strong ties* or *bonding social capital*. Conversely, the bulk of Masisiens' social circles (and thus their support networks) are composed of ‘ordinary’ friends. These are more reserved relationships premised on mutual sharing, with high potential for disappointment. As one participant put it:

*Ordinary friends are not always available. They will not spend their money for you unless you have something to give back*.^13^

Many ordinary friendships are (or start as) locationships with recent strangers. Indeed, as noted, Masisiens are so dependent on these relationships that when people arrive somewhere new, the identification of potential friends is a priority. One man remarked:

*The first thing I do when I arrive somewhere is make friends. It is they who will help me find work*.^14^

The value of these relationships is exemplified by a list of assets and their origin collected in the hut of an IDP family (see[Table disa70055-tbl-0001] Table 2).^15^

**TABLE 2:** List of assets and their origin from the hut of an IDP family.

Asset	Origin
Pots	Gift from a friend
Plates	Gift from a friend
Hoes	Found in another's field
Watering can	Gifted from a friend
Machete	Gift from a friend
Cups	Gift from a friend

**Source:** author, compiled using HH observation data shared by International Alert, N. Kivu, 2015–16.

While intimate friends' social circles overlap, this is less likely among ordinary friends, meaning that the latter connect people to others they may never meet, but who may still be useful (Granovetter, 1983). Differences (social, economic, place) only increase the potential benefits of such relationships. As already highlighted, the literature calls these *weak ties* or *bridging capital*. While such theories are analytically useful, they fail to capture the ebb and flow of relationships, including people's ability and willingness to assist. These are contingent on an unstable blend of personal situation, the internal dynamics of the relationship, and external factors. Depending on circumstance, ordinary friends in Masisi can (and regularly do) provide the type and level of support more usually seen between strong tie friends. Generally, such support is time‐bound, but, when repeated, it rapidly cements intimate locationships. It is this chameleon‐like aspect of ordinary friendships that Masisiens prize most. One man said:

*One of my close friends is my neighbour. There is no family tie between us, but we see each other regularly. We only met a year ago, but he has really helped me, not only in the good moments but also the bad. He lent me money when I was sick and helped me out again to rent a field*.^16^

Their importance is mirrored in the effort people make to find new friends and maintain them. Another man recalled:

*We met five years ago… at work… the boss wanted to fire me, but this friend pled for me and I was kept on. He really helped me out… We don't see each other every day anymore, but we make sure we meet at least once or twice a week. Neither of us have telephones or we could call each other*.^17^

For those who do have mobile telephones, friendships are sustained over a long distance. This allows support networks to cast a much wider net than before. Even when friends disappoint, people rarely ditch them. They may become useful again, and, in conflict, it is ill‐advised to make enemies.

#### 3.1.2 Friends for ‘every eventuality’

Masisiens work hard to have ‘friends for every eventuality’.^18^ They actively cultivate new relationships—as underlined in the quote above: ‘Wherever I end up I try to make friends quickly, these are the people who'll help me’^19^—and nurture as large, socially diverse, and geographically broad a support network as possible. These characteristics (size, diversity, breadth) reflect Masisiens' views of their principal risks: poverty, ethnically targeted violence, and a frequent need to move.^20^

A large support network attenuates the effect of poverty as a person is more likely to receive a little support from many, rather than a lot from a few. This is particularly true in conflict, where a friend's ability to help can change quickly. The size of people's support networks tells us something about their overall vulnerability. Poorer, more socially vulnerable people (displaced, women, youth) have the largest networks and work the hardest to make new friends. This complements research that notes the relationship between poverty, social vulnerabilities, and larger support networks (Kebede and Butterfield, 2009; Desmond, 2012).

Masisiens also seek out friends who are socially different, as they consider such relationships to be protective. The most sought after are friendships with ethnic others. As one woman noted:

*Having friends who are different is good. I live in a mixed area and have Hunde friends. If something is about to happen, they warn me. I do the same for them*.^21^

Also especially desirable are relations with individuals who hold positions of power, but these are hard to make. Masisiens support networks may be ethnically diverse, but their relationships are almost exclusively with their socio‐economic peers. This limits the effectiveness of people's support networks.

**FIGURE 1:**
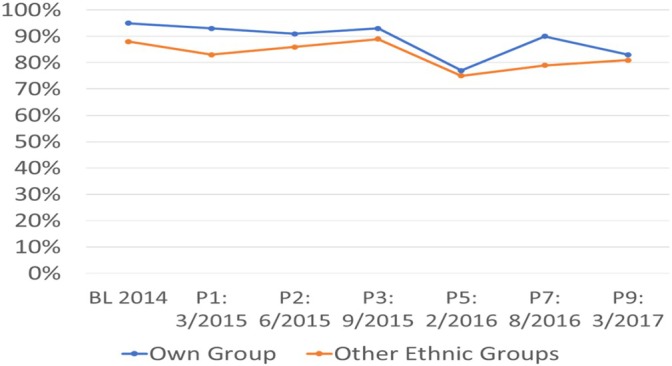
Perceptions of inter‐ethnic social relations, very good to good, Masisi, 2014–17 **Note:** BL = baseline. **Source:** author, compiled using HHI data on https://www.peacebuildingdata.org/studies (last accessed on 24 April 2026).

Conversely, it is relatively easy to make ethnically different friends of the same socio‐economic class. Despite the DRC's ethnicised politics and Masisi's history of ethnically‐targeted violence, social relations between ethnic groups are surprisingly good. This finding complements HHI data which report that Masisiens are ‘very comfortable’ to ‘comfortable’ living in the same village (95 per cent) and in the same household (93 per cent), as well as working with (95 per cent) and even marrying (85 per cent) ethnic others (Vinck and Pham, 2014). Crucially, these percentages track peoples' comfort with their own ethnic group, and do not fluctuate significantly over time, including during periods of increased armed group activity (see Figure 1).

Lastly, Masisiens move frequently for a variety of reasons. Having friends from different places means access to the introductions necessary to make new ordinary friends quickly.

#### 3.1.3 Maintaining friends: the power of sharing

Masisiens believe that ‘you have to give, to be given to’—witness the provenance of an IDP family's assets in Table 2.^22^ Ordinary friendships are cemented through regular contact, small acts of sharing, and, when possible, help in times of need. This ensures the goodwill needed to be supported in turn, but sharing also improves security. As Naureen Fatema and Shahriar Kibriya (2023) find, elite ‘givers of great dinners’ may ‘know few enemies’, but research shows that even small‐scale sharing (particularly of food) reduces low‐grade interpersonal conflict, making people's immediate environment safer. Masisiens recognise that their friends are in the same economic situation and so they do not expect them to respond to every request for support. Occasional refusals are assumed and softened by myriad minor acts, such as braiding a neighbour's hair, providing childcare while a mother works, and sharing a tool or a meal. Yet, a person still needs to respond positively more often than not. For Masisiens, a ‘good’ person is a generous one.

#### 3.1.4 Making friends with strangers: associations, safe space, and reputation

Making friends with strangers is risky, and even more so in conflict. Masisiens minimise this by forging many of their new relationships in three settings: within their neighbourhood; at work; and while participating in both formal and informal associations, such as the church, rotational credit group, and clubs for youth and women. By encouraging proximity and regular exchanges, these locations provide space in which to build trust, while the information that flows across members' networks increases peer pressure and discourages misconduct. Masisiens believe that formal and informal associations are one of the safest places to meet and make friends with strangers. This is due to associations' internal governance structures, the minor penalties they impose for a variety of infractions, and the potentially severe repercussions of a ruined reputation. As one interviewee said:

*We live together and know each other. If I want to join a branch in another village, I need to take a character reference from my [association] branch president. In it, he will explain why I am moving, what I am like. If I don't have a written reference or have lost it, the new president can call up my old one to ask. Also, branch presidents keep in regular contact. One of the main bits of information they share is information about difficult members. Church congregations work the same way*.^23^

This makes Masisiens keen association members and active participants. They often belong to more than one simultaneously, despite the fact that such bodies frequently fold.

I define associations broadly.^24^ In Masisi, they range from a few local NGOs and larger, state‐registered entities (a minority) to smaller, mostly unregistered groupings (the majority).^25^ The latter include friends who help each other at planting and harvesting times, as well as more formal groups from within church congregations and among work colleagues. These tend to organise around credit and saving schemes, or a variety of livelihood activities.

Around 97 per cent of my interviewees were active in various church groups and had joined a rotational work group (87 per cent) during planting and harvesting. More than half (60 per cent) partook in credit and saving schemes and 40 per cent were members of other types of associations, such as formal or informal livelihood, social issue,^26^ or market‐based groups. Using a more formal definition, HHI found that 48 per cent of Masisiens were members of at least one association, with 68 per cent of members attending two or more meetings a month (Vinck and Pham, 2014). To explain their enthusiasm for associations, interviewees pointed out that ‘you can't kill a louse with one finger’ or, more prosaically, ‘in unity lies strength’.^27^

Through their activities, associations aim to improve members' material and financial well‐being, as well as offer ‘insurance’ against the unexpected. One participant noted:

*When there is fighting people come to Goma [the provincial capital]. If they are members, they will come to us. Recently a number arrived. Some had their card, but many had lost it. I called the presidents of their branches to confirm their identity, then we integrated them into our branch. Also, if they have lost stock, we extend a small loan to begin again*.^28^

This loan can be given directly, from one individual member to another, or institutionally via required contributions to a communal cashbox. To be effective, though, these funds need to be replenished. For smaller associations with poorer members, this is difficult.

Besides insurance, associations are protective in other ways. They use the personal networks of leaders and members for information and to exert influence. In Masisi, larger associations successfully lobby state officials and armed groups to reduce ‘taxation’. When a member (or someone in their family) is abducted or arrested arbitrarily, associations utilise their networks to secure their release. In members' eyes, these functions give association leaders the authority to sanction and threaten reputational damage so as to manage members' behaviour. This improves security within the association, making them a relatively safe space in which to meet and make friends with a stranger. Indeed, members feel that associations create a psychologically comforting ‘sense of normality’ in distinctly abnormal circumstances.^29^

Nevertheless, most associations provide neither effective nor sustainable social protection. Rather than institutional support, it is members who become friends that help each other most. Moreover, many associations shut down within a couple of years. What survive them are a person's reputation and the relationships made while participating. It is these elements that ensure Masisiens remain eager participants.

#### 3.1.5 Reputation and trust

In conflict, material and financial assets are at risk, as they are hard to hide, inconvenient to move, and easily stolen. They make people targets and so Masisiens would rather not invest in them. The little that they have, they avoid keeping in one place. Enter reputation. In Masisi, this acts as an intangible currency ‘spendable’ on a whole variety of goods and services, such as influence, shelter, food, credit, and jobs. As a number of participants remarked:

*When I had difficulties, the notables and authorities helped me because they know me well*.^30^

*As I had a good reputation, we were welcomed by family friends we didn't know. In the end they hosted us for six months until things quietened down*.^31^

*If we know someone is honest but doesn't have the necessary capital, the Likirimba [informal credit and savings group] will accept them if we think our support will make their income large enough for them to contribute. To help get them up to speed, we serve them first*.^32^

Reputation is relational: it is built and maintained through regular exchange and small acts of reciprocal support. When people arrive in a new place, they use their networks to broadcast (and confirm) reputation. This makes settling in easier and faster. Any Masisien expects the ‘buyer’ to check with an association/community leader or a mutual friend about their ‘spending’ reputation. Nowadays, mobile telephones/SIM (Subscriber Identity Module) cards mean people can ‘spend’ reputation further afield than before.^33^

Associations provide the perfect space in which to build and broadcast reputation. As one study participant underlined:

*If a person has an association card, you know they have a good reputation and that they are a person you can trust*.^34^

But it is a gamble. A poor reputation is hard to outrun, and ostracism has serious consequences. A woman explained:

*If voice gets around that you create problems, your reputation is ruined, and people will not hire you [or let you join another association]*.^35^

Trust and reputation are intertwined. However, trust in Masisi is not robust; rather, it is pragmatic and replete with reservations. Masisiens believe that trust is born of ‘love and money’, where sharing fuels the ‘love’ (willingness/inclination) to reciprocate.^36^ People's need is so great that it forces them to trust, often with dire consequences. Associations see the problem and work hard to build trust between members. They achieve this by getting members to collaborate, contribute to the cashbox, and support each other directly. Associations shame and sanction members who do not meet their obligations. One participant emphasised:

*We check reputation, but we also have prospective members pay USD 10 upfront and later a further USD 20 before admitting them to full membership. This gives us time to watch them work and see if they are serious or not… We also apply sanctions [fines, temporary suspensions] when members are arrogant, do not follow regulations, or accept decisions taken by the leadership committee… [These things] help us trust each other*.^37^

Yet, not all associations build trust. The least successful are those founded and funded by outsiders. These are also the quickest to fold. It is the smaller associations of prior friends and the larger, grassroots associations whose members regularly replenish the kitty that build trust best. Where trust is weak, reputation bolsters it. Ultimately, whether used as an unstealable currency ‘spendable’ on a wide variety of goods and services or as a proxy for trust, it seems that reputation, rather than direct social protection, is associations' most useful individual outcome.

#### 3.1.6 Patterns of support

Although this subsection provides an initial overview and analysis of the relations and support networks on which Masisiens rely, variables such as a person's sex, age, physical ability, social and socio‐economic status, area of residence, and social affiliations (including ethnicity, church, and school) influence their configuration and effectiveness. More research is needed to tease out these nuances, but my data suggest some initial trends: notably, wealthy, often older, elite men have large circles of acquaintances but rely most on small, influential support networks. These connect closely with economic and political power and tend to be geographically diverse. Furthermore, while elite men's *acquaintances* may be ethnically diverse, their support networks appear to be less so.

Poorer young men have among the largest, most ethnically diverse and geographically widespread support networks. These relationships develop principally among work colleagues, neighbours, and school fellows. The size, ethnic and geographical diversity of young men's support networks mean that they are able to provide a degree of assistance, but the lack of socio‐economic diversity means that its quantity and reliability are limited.

Younger and family‐age women build their support networks primarily through social exchanges in fields, in local markets, and at church. They are most likely to have a male relative among the members of their support network. Women's support networks tend to be among the most ethnically diverse. However, as they are composed mainly of women of similar socio‐economic status, the material and financial support that they offer is often limited. Lastly, the near destitute (a minority) have the smallest circle of friends and the most limited and least diverse support networks. These are mostly ‘friendless’ older people and widowed/abandoned women‐headed households.

For most Masisiens, the social protection that their support networks provide is limited and never guaranteed. This is due in part to poverty, but also to the relational nature of resilience. Although relationships and networks are people's safest and most effective prospects of resilience, they are inherently unstable. Their ability to deliver hinges on personal and relational dynamics as well as on external factors often beyond anyone's control. Their best outcome is more the reputation they afford, rather than the social protection they furnish directly. Thus, while Masisiens may achieve *moments* of resilience, sustainable resilience remains elusive for most.

### 3.2 Webs of governance: support networks, security, and coercive social control

Support network research tends to focus on the provision of informal social protection, while the cumulative effects of people's intersecting networks remain understudied. Yet, scholars of genocide and civil war intimate that leaders and their communities use networks to resist violence and note the importance of social control (Fujii, 2009; Kaplan, 2017; Krause, 2018; Howe, 2023). My data suggest that in Masisi, community leaders not only use these relationships to broker temporary ‘zones of peace’, but also that they leverage their involvement in local support networks, particularly different types of associations, to manage internal violence and improve localised security. However, community leaders are helped in this respect by the coercive potential of support networks, which in turn is amplified by Masisiens' fear of ostracism. Like trust, social control in Masisi is premised on need. Paradoxically, were this to lessen, leaders' social control might diminish, with consequences for local security.

#### 3.2.1 Community governance and internal security

Most community leaders in Masisi are older, elite men. Their status comes from (relative) wealth, social position, and their ability to attract followers. Leaders' support networks may be small and select, but their social circles are among the largest and most socio‐economically diverse. These small‐scale ‘big men’ (Utas, 2012) run the gamut from pastor to warlord and wear multiple hats. They may be members of customary authority and have close ties to armed groups, but they almost always head different support networks, including the largest and most powerful associations. As one underlined: ‘we don't need to lobby the state, we are the law’.^38^

If all associations provide a degree of internal governance, bigger associations have evolved into parastatal institutions that govern in complement to state and customary authority.^39^ These include larger livelihood associations, market committees and market‐based associations, churches, and larger local NGOs. Registration as non‐profits (Association à but non lucratif) makes these actors members of Congo's formal civil society, which has a cosy relationship with the state.^40^ In Masisi, lodging a complaint with state officials is discouraged. Officials expect customary authority and associations to govern community behaviour. When differences arise between members of different associations, it is association leaders who adjudicate disputes. If members go to the police, ‘it comes right back to us, and we sanction them for it. The police know it needs to go through us first… You don't air dirty laundry in public!’.^41^

Together with customary authority, leaders of large associations sit at the apex of a network of hierarchically ranked association leaders. They use these connections (and their subnetworks) to govern through social control. For Masisiens, the outcome is an increased sense of security. HHI's data corroborate this finding. At the time of fieldwork, 75–83 per cent of HHI's respondents reported feeling *safe* or *very safe* while conducting regular activities in their communities during the day. The advent of night reduced this to 53–64 per cent. The percentage is high given the circumstances, but the situation is precarious. Shortly after one of my visits, Masisiens' perception of security decreased hugely. In the months between July and December 2017, the daytime sense of security fell from 71 to 58 per cent. By the end of the year, only 29 per cent of Masisiens reported feeling *safe* or *very safe* when moving around at night. This may reflect new uncertainties unleased by President Joseph Kabila's postponement of the 2016 elections. By December 2018, as his successor, Félix Tshisekedi, prepared to be sworn in, Masisiens' sense of daytime security had seemingly recovered, reaching 81 per cent (Vinck et al., 2019).

#### 3.2.2 Resisting external violence

Being perceived as the guarantors of internal security gives community leaders the legitimacy to resist external violence. These efforts are usually spearheaded by small groups of senior leaders and relevant community members.^42^ The length of the conflict, along with the cyclical nature of armed group membership, mean that leaders and community members often have close personal ties to individual fighters and the leadership of armed groups (Vlassenroot, Mudinga, and Musamba, 2020). When needed, such connections allow community leaders to reach out to, and negotiate with, armed groups. Yet, this should not be taken for granted. There are many reasons why community leaders sometimes negotiate and sometimes do not. Even when they do, success is not guaranteed or may not encompass everyone. Community leaders not only capitalise on their authority to maintain internal security and broker local peace, but they also use it to commodify protection. Many utilise their authority to extend protection patronage and extract financial benefits, which, in turn, increase their influence. It is evident, therefore, that protection is not just a public good, but that it can also be a socially and economically lucrative private one (Verweijen, 2023). This highlights how apparent short‐term gains—in this case, increased security—are achieved at the cost of reinforcing hierarchies that entrench the status quo.

## 4 CONCLUSION

So, is resilience in Masisi relational? Certainly, those with few friends quickly succumb to the challenging circumstances in which they live. Not only are social networks key to understanding resilience in conflict, social networks *are* resilience. This is because relationships are people's most flexible and safest asset. These qualities also make them their most reliable resource. Yet, despite the web of support on which most people call, resilience in Masisi is fragile. Poverty, uncertainty, and movement constrain the assistance that connections provide, while relational dynamics and external conditions make it unpredictable. Therein lies the paradox: resilience in conflict may, by necessity, be primarily relational, but its reliance on relationships also limits it and makes it unstable.

Masisiens mitigate these risks by cultivating large, ethnically diverse, and geographically broad networks of ‘ordinary friends’, many of whom are recent strangers. People nurture such friendships because they believe that they provide the most effective, overall support. But a person's capacity (and willingness) to support fluctuates depending on personal circumstances, the internal dynamics of the relationship, and external factors; while gender, age, class, and (dis)ability shape support networks in ways that remain understudied. Perhaps most importantly, as support is premised on exchange, Masisiens' lack friends who are socio‐economically better off. Instead, they rely on friends from similar socio‐economic backgrounds. This limits the level, reliability, and effectiveness of the support people can expect.

Moreover, when Masisiens (mostly men) do forge relations with those more powerful than themselves, they tend to be based on a different understanding of mutual reciprocity. These are hierarchical, clientelist relationships, where support and protection are often commodified and used as patronage. It is at this point that people's everyday support networks intersect the vertical, more political networks that link local micro dynamics and macro drivers of conflict, but this is a topic for another article.

Masisiens invest great effort in making new friends. Associations provide a safe space in which to do so. Although most of these bodies fold, the friendships they nurture often outlive them. The relational aspect—rather than the institutional social protection offered—is why Masisiens join associations in droves. It is also why people use them to build and broadcast their reputation. In Masisi, reputation has become a vital currency, ‘spendable’ on a range of goods and services, and, in a context where trust is weak, Masisiens use reputation to boost it.

However, Masisiens support networks also serve a less visible function: they are important sources of alternative governance. This helps to improve local security and gives community leaders the legitimacy to regulate internal violence and stem its encroachment from outside. At their most basic, support networks are premised on sharing. Secondary research suggests that sharing reduces conflict between community members, while my own data show how intersecting support networks weave a dense web of information exchange that discourages bad behaviour and increases people's sense of safety.

At a more structural level, Masisi's plethora of associations creates a supra network of hierarchically ranked leaders. At its apex sit customary authority, community leaders, and large association leaders (it is not uncommon for a community leader to hold two if not three of these positions). Community leaders use their connections (and associations' subnetworks) to enforce social control and govern the community. State officials capitalise on this, counting on customary authority and association leaders to adjudicate non‐criminal disputes between members. For ordinary Masisiens, this augments their sense of safety and affords community leaders considerable authority. Community leaders can capitalise on this to resist external violence. These efforts are usually headed by a small group of the most important leaders and relevant community members. The length of the conflict, along with the cyclical nature of armed group membership, means that leaders and community members frequently have close personal ties to individual fighters. Leaders use these connections to reach out to armed groups and negotiate with them. Nonetheless, these efforts are not consistent and cannot be taken for granted. Community leaders do not always negotiate; and even when they do, success is not guaranteed. Although the support they provide may be limited, my interviewees believe that their everyday support networks ‘improve their lives’ and create a comforting sense of ‘normality’ in distinctly abnormal circumstances.

This article is a first attempt to tease out some of the intricacies of people's support networks and to explain how and why resilience in and to conflict is primarily relational. This nuances and extends understanding of resilience and coping in conflict, as well as the role that relationships and networks play in assisting and protecting individuals and communities. The paper also offers an initial analysis of the cumulative social‐political effects produced by overlapping support networks. This provides glimpses of the paths that link the individual to the collective and the private to the political, revealing intriguing avenues for further research.

The implications of such findings, however, are not only academic. For more than a decade humanitarian policy and programmes have been intrigued by social networks and their potential to support communities living with conflict. Yet, relational approaches have made few inroads in practice. Such findings urge practitioners to think through what centring social relations in practice might look like. Indeed, unpicking the relationships and power dynamics that underpin the support systems on which people rely, and being aware of their aggregate effects, have never been more pertinent. As aid budgets are slashed and the operational capacity of agencies constricts, it is vital that we appreciate how to reinforce positively people's support networks, while taking their power dynamics and cumulative outcomes into consideration.

## CONFLICT OF INTEREST STATEMENT

There is no known conflict of interest.

## Data Availability

The data that support the findings of this study are available on request from the corresponding author. The data are not publicly available due to privacy or ethical restrictions.

## Endnotes

^1^
Zaire was officially renamed the DRC in May 1997.

^2^
Whether migration or displacement.

^3^
Either French, KiSwahili, KiHunde, Kinyarwanda, KiNande, and/or KiNyanga; oftentimes, a mixture of several languages.

^4^
For more information, see https://www.peacebuildingdata.org/studies (last accessed on 24 April 2026).

^5^
Interviews, Masisi, 2015–16.

^6^
Woman, 70, south‐central Masisi, 26 July 2016; and interview, president and member of a formal livestock association, Masisi Centre, March 2014.

^7^
Woman, Lushebere Centre, 25 November 2015.

^8^
Ategeka, man, northern Masisi, 29 July 2016.

^9^
Community and support network leadership, such as of a church, association, clan, and lineage, often intersect.

^10^
Percentages from interviews carried out in Masisi, January–August 2016.

^11^
Man, 34, southern Masisi, 4 August 2016; based on self‐reporting and narratives of specific incidences in which interviewees received support.

^12^
Dismas, 72, man, southern Masisi, 25 July 2016.

^13^
Innocent, 27, man, southcentral Masisi, 5 August 2016.

^14^
Emmanuel, 25, man, northern Masisi, 24–25 July 2016.

^15^
International Alert, north Masisi, November 2015.

^16^
Kingombe, 33, man, southcentral Masisi, 25 July 2016.

^17^
Safari, 29, man, central Masisi, 23–25 July 2019.

^18^
Sadiki, 43, southwest Masisi, 27 November 2015.

^19^
Ategeka, man, northern Masisi, 29 July 2016.

^20^
Between 1993 and 2007, HHI recorded that Masisiens were displaced by conflict an average of 3.7 times (Vinck et al., 2008). My data suggest that the figure is now considerably higher, with people displacing several times a year over shorter distances.

^21^
Marie, 42, woman, northwest Masisi, 20 March 2016.

^22^
Woman, 36, south‐central Masisi, 31 July 2016.

^23^
Key informant interview, northern Masisi, 31 October 2015.

^24^
‘A group of people organised for a joint purpose’ (source: *Oxford English Dictionary*).

^25^
Mostly agropastoral, social issue, or market‐based associations.

^26^
These groups tend to focus on capturing external funds and organise around social issues such as sexual and gender‐based violence or promoting peaceful cohabitation between ethnic groups.

^27^
Association interviews in Goma, 2014, and Masisi, 2015–16.

^28^
FGD, provincial leadership of a large association, AVPS (Association Vendeurs de Poisson Sani/Association of Fish Vendors), Goma, April 2014.

^29^
President and member of a formal livestock association, Masisi Centre, March 2014.

^30^
Donat, 65, man, northwest Masisi, 24 March 2016.

^31^
Theophile, 62, man, northern Masisi, 10 November 2015.

^32^
FGD, a large Likirimba, northern Masisi, November 2015.

^33^
While people may not have the means to have a mobile telephone, they will often have a SIM card that they can put into one to send/receive messages for a small fee.

^34^
Large association member, central Masisi, March 2014.

^35^
FGD, IDP women, Kilimani Camp, Masisi Centre, 26 October 2015.

^36^
Aimant, 25, woman, FGD/Cassava flour sector, central Masisi, April 2014.

^37^
FGD, AVPS leadership and members, Masisi Centre, March 2014.

^38^
Community leader, Masisi, July 2016.

^39^
Except in the principal city, Masisi Centre. Here associations are the only form of alternative governance as custom has no jurisdiction.

^40^
Civil society in DRC is closely tied to the state. Created by the state as a watchdog after the 2006 elections, it is institutionalised and highly regimented. Its members are regulated, activities are state‐sanctioned, and its cadres are connected to state authority. Rather than providing a uniting platform for meaningful negotiation with the state, civil society is at best toothless; at worst, it extends the state's controlling arm (Fontana, 2014, p. 19).

^41^
FGD, Boutiquiere's association (association of shopkeepers), central Masisi, October 2015; Fidel, 71, man, northern Masisi, 30 July 2016.

^42^
That is, non‐leaders who have close connections with armed actors.
